# Do Alu repeats drive the evolution of the primate transcriptome?

**DOI:** 10.1186/gb-2008-9-2-r25

**Published:** 2008-02-01

**Authors:** Araxi O Urrutia, Leandro Balladares Ocaña, Laurence D Hurst

**Affiliations:** 1 Department of Biology and Biochemistry, University of Bath, Bath, BA4 7AY, UK; 2 Computer Research Center of the IPN, Mexico City, Mexico 07738; 3 Department of Computer Engineering at University of California Santa Cruz, Santa Cruz, California 95064, USA

## Abstract

**Background:**

Of all repetitive elements in the human genome, Alus are unusual in being enriched near to genes that are expressed across a broad range of tissues. This has led to the proposal that Alus might be modifying the expression breadth of neighboring genes, possibly by providing CpG islands, modifying transcription factor binding, or altering chromatin structure. Here we consider whether Alus have increased expression breadth of genes in their vicinity.

**Results:**

Contrary to the modification hypothesis, we find that those genes that have always had broad expression are richest in Alus, whereas those that are more likely to have become more broadly expressed have lower enrichment. This finding is consistent with a model in which Alus accumulate near broadly expressed genes but do not affect their expression breadth. Furthermore, this model is consistent with the finding that expression breadth of mouse genes predicts Alu density near their human orthologs. However, Alus were found to be related to some alternative measures of transcription profile divergence, although evidence is contradictory as to whether Alus associate with lowly or highly diverged genes. If Alu have any effect it is not by provision of CpG islands, because they are especially rare near to transcriptional start sites. Previously reported Alu enrichment for genes serving certain cellular functions, suggested to be evidence of functional importance of Alus, appears to be partly a byproduct of the association with broadly expressed genes.

**Conclusion:**

The abundance of Alu near broadly expressed genes is better explained by their preferential preservation near to housekeeping genes rather than by a modifying effect on expression of genes.

## Abstract 2

The abundance of Alu elements near broadly expressed genes is best explained by their preferential preservation near housekeeping genes.

## Background

Repetitive elements constitute 45% of the human genome [1]. With more than 1 million copies (about 10% of the human genome), Alu sequences are the most prevalent repetitive elements [2]. Alus began to spread at the base of the primate lineage about 65 million years ago [3] and inserted at high rates until about 30 million years ago, after which Alu insertion rate was markedly reduced. This translates to 85% of Alus being common to all monkeys [4]. Because they are primate specific, Alus have been proposed to be major players in shaping the primate genome and transcriptome. However, little is known about the impact they have on genome structure and function. Although they are considered genetic 'junk' by some authors [5], others have proposed that they are functionally important [1,6-8]. In a few instances they have been found to have inserted into coding regions of genes, becoming part of the protein coding message [9,10]. Similarly, newly inserted Alu elements may trigger genomic responses such as recombination/replication slippage and CpG methylation, which can lead to gene duplications/deletions and help to produce new alternative splicing isoforms [11,12]. In addition, phylogenetic studies have identified a relation between lineage divergence and increased rates of transposition in primates, prompting the possibility that Alu expansions play a role in speciation [8].

At a genomic level, Alu sequences are not randomly distributed along the genome and are found in higher densities in gene rich regions [13]. Alu sequences are more common in GC-rich genomic domains, which are also the most gene dense sections of the genome [1,2,14]. Almost three-quarters of genes have Alu sequences in their flanking regions [2], placing these repeats in stretches of sequence potentially relevant to gene regulation. Indeed, in our sample we find that Alus are enriched near to genes occupying 18.5% of the sequence (in the 20 kilobase [kb] flanking region of genes), as compared with 12.8% of intronic sequence and just 9.6% of intergenic regions [7]. Perhaps more startling is the observation that Alu sequences are more common in flanking regions of highly expressed and housekeeping genes than in lowly expressed and tissue-specific ones [15-17]. This difference persists even when one takes into account the isochore type in which the genes are residing, suggesting that the Alu enrichment around housekeeping genes is not a byproduct of differences in Alu insertion rates among different genomic compartments [17]. The enrichment is found for both newer and older Alus, although it is more pronounced for the older ones [17]. Likewise, analyses of genes located on chromosomes 21 and 22 revelaed Alu sequences to be unequally distributed within genes serving different cellular functions [18].

What accounts for Alu enrichment near to housekeeping genes? Two broad classes of model can be considered. In the first, Alu sequence enrichment causes an increase in expression breadth, which here we term the 'expression modifier' model. Alternatively, Alu enrichment of housekeeping genes could be the result of a process that is unrelated to the modification of expression profiles, which we term the 'marker model'. This marker model may be neutralist or selectionist.

In support of the first possibility, Alu involvement in regulation has been demonstrated for a handful of genes through experimental approaches [6,19-26]. Moreover, several viable mechanisms have been proposed by which Alu might influence gene regulation, causing them to be more broadly expressed. CpG islands are stretches of DNA with a greater than average frequency of CpG dinucleotides [27,28], and they have been found on promoter regions or first introns of over half of human genes [29-32]. CpG islands are more common in the upstream region of genes expressed in many tissues [28,29]. Importantly, Alu sequences are unusually rich in CpG dinucleotides [33,34], suggesting the possibility that Alu sequences contribute to increases in the breadth of expression of genes through introducing CpG islands. Alternatively, localized GC content in the vicinity of genes may make chromatin opening easier and hence aid transcription. Alu insertion may thus modify local GC content. This is akin to Vinogradov's idea of a 'gene nest' [35]. Finally, known regulatory sequences that respond to hormones, calcium, and transcription factors have been found in consensus Alu sequences and have been shown to regulate transcription in some genes (for review [7]). A final possibility, for which we know of no evidence, is that Alu insertion might disrupt a tissue-specific promoter element, causing the gene to be more broadly expressed. With the exception of this latter possibility, all of the other models propose a gain of function concomitant with Alu insertion that would be specific to Alu (any repetitive element could in principle disrupt a tissue-specific promoter). In this regard, all three models have the potential to explain why Alu in particular among the repetitive elements are unusual in being enriched near to housekeeping genes.

Taken together, the findings mentioned above are then consistent with the possibility that Alu sequences are not just a major player in the evolution of the primate genome but also an important factor in shaping gene regulation during primate evolution [6,7,12,36,37]. As for the 'marker model', this would require that some insertion/expansion/conservation bias not causally related to gene regulation is taking place and accounts for the unequal distribution of Alus near to genes with varying expression profiles. Eller and coworkers [17] have suggested the neutral possibility of Alu sequences accumulating around housekeeping genes because of the deleterious effects of excision by recombination of neighboring Alu sequences. There is also a selectionist alternative that is consistent with the marker model. According to experimental findings, increased short interspersed nuclear element (SINE; the repeat family that includes Alus) transcription is observed under particular stress conditions [38-41], coinciding with expression of heat shock proteins [41-43] and leading to speculation that they could be playing a role in cell stress recovery, although it is not clear what this role might be. In any case, under the marker model Alus would accumulate near to highly expressed and/or housekeeping genes, but they do not modify their expression breadth.

Here we attempt to distinguish the expression modifier and marker models. Using three separate transcriptome data (microarray [44], Serial Analysis of Gene Expression [SAGE] [45], and Bodymap [46]), we first investigate the relationship between Alu content in flanking regions and gene activity at a genomic scale. In particular, as housekeeping genes tend also to be highly expressed (they are expressed at a high rate in many tissues) and to be enriched in GC-rich domains, we consider whether the enrichment near to housekeeping genes is actually better explained as enrichment near to highly expressed genes or simply as enrichment in GC-rich domains. We find that the enrichment is best explained as being in the vicinity of housekeeping genes. Is it the case, then, that Alu are responsible for an increase in breadth of expression of genes in their vicinity? To distinguish between the models we also consider whether any enrichment is more profound 5' than 3' and whether the Alus are especially prevalent in the more immediate vicinity of genes (for instance, near to the transcription start sites, as predicted by the CpG island model). We then investigate whether Alu repeat insertions have played a relevant role in the evolution of increased gene expression breadth using a comparative genomics/transcriptomics to examine two independent expression datasets: microarray [44] and Bodymap [46]. The role of Alus in other forms of expression divergence is also examined.

## Results

### Alu content is enriched near broadly expressed genes not highly expressed genes

**Figure 1:**
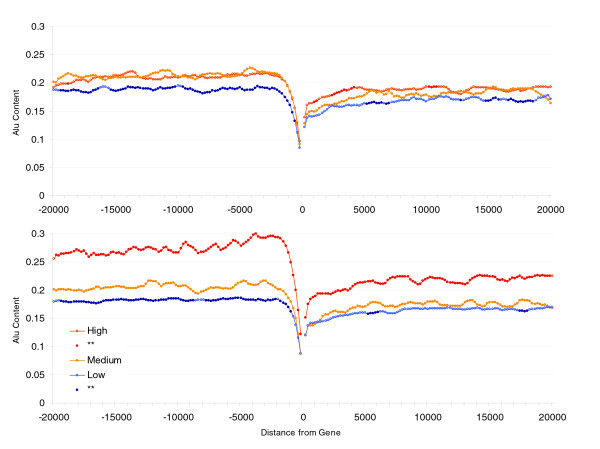
Alu content in flanking regions of human genes (20 kilobases) and expression profiles. Groups represent the 20% most highly ('High'), least highly ('Low'), and the medium expressed genes ('Medium') for peak (top panel) and breadth (lower panel). Points for high and low groups significantly different from medium expression levels (Student's *t*-tests using Bonferroni correction) are represented by closed circles. Each point represents the Alu content in sliding windows of 1 kilobase (moving 200 base pairs at a time).

We start by establishing that the important pattern, namely that the association between Alu presence and expression parameters, is real and not explained by correlation with some other variable. To this end, using three separate sources for expression profiles (see Materials and methods, below), we ranked all genes according to two indices of gene activity: breadth (number of tissues in which a gene is expressed) and peak expression (highest expression in any tissue). Considering the top 20% (those more highly/broadly expressed), the bottom 20% (those more lowly/narrowly expressed), and the middle 20%, we found that broadly expressed genes exhibit an average 10% increase in Alu content on their flanking regions compared with genes with a narrower tissue distribution. Although several authors have reported a relation between Alu content and expression profiles, none has attempted to quantify the variance in expression data that is being explained. To assess the actual predictive power of Alu content on expression profiles, we conducted a regression analysis on the 4 kb section that exhibits the greater differences among groups (2 to 6 kb from start/end of transcription). For breadth of expression, the correlation with Alu content explains at most 5% of the variance (microarray/SAGE/bodymap data [*n *= 15,147/13,622/10,281]; upstream: *r *= 0.160/0.225/0.191 [*P *< 0.001 for each]; downstream: *r *= 0.107/0.156/0.096 [*P *< 0.001 for each]). The quantitative measure of expression (peak expression) has a weaker relation with Alus (microarray/SAGE/Bodymap data [*n *= 13,134/13,622/10,281]; upstream: *r *= 0.041/0.079/NS [*P *< 0.001 for microarray and SAGE, NS for Bodymap]; downstream: *r *= 0.050/0.081/NS [*P *< 0.001 for microarray and SAGE, NS for Bodymap]; Figure 1 and Additional data file 1). The relation between Alu content and the quantitative measure of expression is no longer significant when peak is corrected by breadth of expression while the opposite does not occur (except for SAGE data, for which a significant correlation explaining 0.1% of the variance is still observed with downstream Alu content).

### Alu content enrichment near broadly expressed genes is not a side consequence of co-variation with GC content

The above findings suggest that the link between expression and Alu content in flanking regions is mostly due to a primary correlation between Alu and expression breadth. This is potentially consistent with a model in which Alus are indeed involved in gene regulation. However, the relationship with expression breadth might simply be a byproduct of other, independent interactions of sequence parameters with gene activity and Alu density. GC content is thought to be related to gene activity [47-53] (but see [54,55]) and with density of Alu sequences [1,14]. Therefore, it is possible that both broadly expressed genes and Alu repeats concentrate in regions of high GC content. To investigate this possibility, we corrected Alu content in flanking regions for the relationship with GC content and then we reassessed the relationship with expression breadth (see Materials and methods, below). We found that, after correcting for the relationship of intergenic GC with Alu content, Alu content remained significantly higher among broadly expressed genes than among lowly expressed genes in both upstream (microarray/SAGE/Bodymap data [*n *= 15,147/13,622/10,281]; *r *= 0.163/0.200/0.205 [*P *< 0.001 for each]) and downstream (microarray/SAGE/Bodymap data [*n *= 15,147/13,622/10,281]; *r *= 0.123/0.141/0.090 [*P *< 0.001 for each]) regions. Hence, the effects are not explained by co-variation with GC content.

### Alu content is enriched both 3' and 5' of broadly expressed genes

The several ways in which Alus could be affecting expression breadth predict different patterns of Alu enrichment 5' and 3' of housekeeping genes. First, if Alus are providing CpG islands that are relevant to gene transcription, then we expect Alus to be enriched near to the transcription start site (TSS) and to exhibit no tendency to accumulate 3' of housekeeping genes. Likewise, if Alu are providing novel transcription factor binding sites or other regulatory elements (or disrupting tissue-specific control elements), then they should be abundant 5' but not 3'. By contrast, if Alus are affecting overall GC content, and as such altering chromatin structure to render housekeeping genes more accessible for transcription, then both 5' and 3' enrichment is expected and we need not predict enrichment near to the TSS.

Under the marker model predictions are not so clear. In the simplest case, in which insertion is simply into open chromatin near to transcriptionally active genes, we might expect enrichment 5' and 3'. However, close analysis of several classes of retroelement and transposon reveals that insertion is biased to the 5' end (for instance, see [56-59]). Hence, this model could be consistent with many possibilities and is hence hard to falsify with this test, without better knowledge of the insertion biases of Alu and subsequent biases in their evolution. However, enrichment 3' more than 5' is not obviously predicted by this or any model. Note, though, that a simple insertion bias model is probably not adequate on its own, because enrichment of Alu sequences in GC-rich stretches of the genome is probably not the result of insertion bias, as Alus insert preferentially on AT-rich regions [1,60,61] (but see [62]).

**Figure 2:**
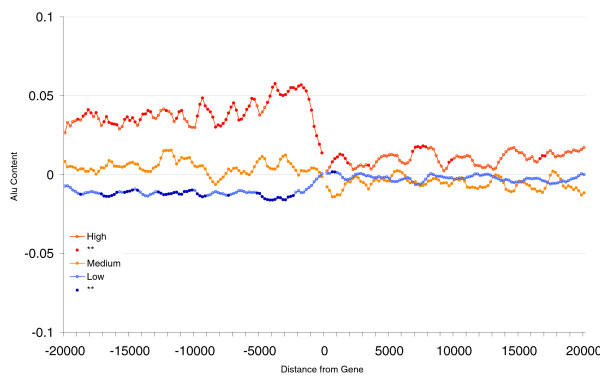
Correction for regional Alu density. Shown is the Alu content in flanking regions of human genes (20 kilobases) and expression profiles correcting for regional Alu density. Each point represents the Alu content in sliding windows of 1 kilobase (moving 200 base pairs at a time) after correcting for regional Alu density (Alu content in opposite flank of gene) through regression analysis (see Materials and methods). Groups represent the top 20% of genes with highest ('High'), 20% with the lowest ('Low), and 20% of medium ('Medium') breadth of expression. Points for high and low groups significantly different from medium expression levels (Student's *t*-tests using Bonferroni correction) are represented by closed circles.

In Figure 2 we can observe that the difference in Alu content between broadly expressed and more tissue-specific genes is greater for the 5' flanking region than the 3'; however, the difference is significant for both flanks. There is hence both a regional effect and a 5'-specific effect. To remove any regional effect we corrected Alu content on each flanking region for the Alu content on the opposite flanking region (see Materials and methods, below) and repeated the comparison of Alu content among the gene groups of different expression breadths and level. Results from regression analyses on the whole sample show that the difference in Alu content for broadly and more tissue-specific genes is largely unchanged for the upstream (5') region (microarray/SAGE/Bodymap [*n *= 15,147/13,622/10,281]; *r *= 0.128/0.164/0.165 [*P *< 0.001 for each]), whereas the difference in Alu content for the downstream (3') flanking region is diminished but the relation does not disappear completely for two of the three datasets tested (microarray/SAGE/Bodymap [*n *= 15,147/13,622/10,281]; *r *= 0.47/0.049/NS [*P *< 0.001 for microarray and SAGE, and NS for Bodymap]). We therefore conclude that the relation between breadth and Alu content is higher for the 5' region, but there is also a regional component. The regional effect would argue against the 5' promoter and CpG island models. The 5' enrichment controlling for any regional effect is contrary to the chromatin model. A mixed model cannot be excluded. However, given some not inconsiderable uncertainty in gene annotation and the possibility that the 3' end of one gene may be the 5' end of another, definitive conclusions are hard to draw from these findings.

However, what does seem clear is that the Alus are specifically avoided in the vicinity of the TSS. In addition, Alus, although CpG rich, appear not to share the qualities of CpG islands that are found on proximal promoters of genes [32,63]. Notably, unlike CpG islands in the near proximity of genes, Alu CpG repeats appear to be ubiquitously methylated [64]. For these reasons, we reject the modification of CpG islands model. The marker model may be consistent with the patterns, especially because a 5' insertional bias has been described for some retroelements [56]. If we assume that Alu insertion is possible near TSSs, then their dearth near to TSSs implies purifying selection against such insertions, probably because they disrupt expression.

### Alus accumulate near to housekeeping genes but they do not alter expression breadth

**Figure 3:**
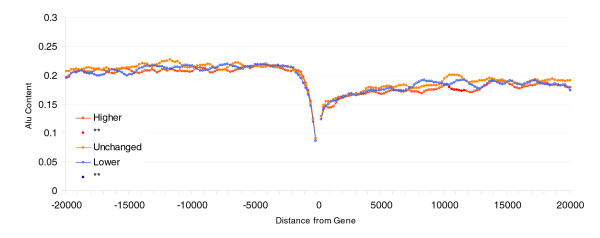
Difference in breadth of expression in human-mouse orthologous genes. Shown are Alu content in flanking regions of human genes (20 kilobases) and difference in breadth of expression in human-mouse orthologous genes. 'Higher' refers to the top 20% of human genes with expression in a higher number of tissues than their mouse counterparts; 'Unchanged' includes the middle 20% of genes in the distribution; and 'Lower' refers to the 20% of genes with lowest breadth of expression with respect to their mouse orthologs.

To investigate whether increased Alu content near broadly expressed genes is due to the boosting effect on expression breadth of Alu insertions, we conducted a comparative transcriptome analysis. Because the majority of Alu sequences are common to all primates, it is adequate to address this issue using a nonprimate species to compare gene activity. By using a nonprimate species (which therefore would not have Alu in its genome), we also eliminate the errors derived from the mis-identification of lineage-specific Alu insertions that would occur with use of primate species. The mouse transcriptome, after that of human, is the best characterized. We therefore calculated the difference in breadth of expression between pairs of human and mouse orthologs and compared these differences with Alu content of flanking regions. Do then Alu-rich genes have greater breadth than their mouse orthologs? The results here are contradictory but suggest at the most that Alus explain only a tenth of 1% of the variance (microarray/Bodymap data [*n *= 11,275/8,179]; upstream: *r *= 0.005/0.039 [*P *NS for microarray and *P *< 0.001 for Bodymap]; downstream: *r *= 0.003/0.031 [*P *NS for microarray and *P *= 0.005 for Bodymap]; Figure 3 and Additional data file 2). These data hence provide no strong support for the hypothesis that Alu accumulation explains much of the increase in expression breadth.

This finding is suggestive of a scenario in which Alus insert or accumulate near to genes that already have high breadth of expression. Because Alu is human specific, we could provide direct support for this model by showing that expression of nonprimate genes predicts Alu content of human orthologs. In support of this alternative position, we find that breadth of expression in the mouse genome well predicts Alu content of the orthologs in the human genome (in mouse, microarray/Bodymap data [*n *= 11,275/8,179]; upstream: *r *= 0.142/0.218 [*P *< 0.001 for both]; downstream: *r *= 0.093/0.115 [*P *< 0.001 for both]). This indicates that genes that have always been broadly expressed are those that are enriched for Alu rather than those that have had their expression breadth increased. Note also the strength of this effect. The upstream correlation we observe with bodymap data is unusually strong. Given that this cannot be due to causative effects of Alu, this provides strong support for the marker model.

To further test whether this is indeed the case, we took all human housekeeping genes in our sample and then partitioned them into groups according to the expression pattern of their orthologous genes in mouse. We then compared the Alu content of housekeeping genes in human that were also housekeeping genes in mouse (*n *= 841) against those genes that were housekeeping genes in human but tissue-specific in mouse (*n *= 128). In the first group, the most parsimonious assumption is that the gene was a housekeeping gene before the two lineages split. In the second group, the gene either lost its broad expression in the mouse lineage or became expressed in more tissues in the human lineage; we can assume that about half of all cases fall into each category. Therefore, for the first group human genes would for the most part have been broadly expressed during the evolution of the primate lineage. In the second group, however, some proportion of genes would initially have been tissue specific and gained their housekeeping status later in the evolution of the primate lineage. If Alus are merely accumulating in flanking regions of housekeeping genes, then we would expect them to be more prevalent in the first group than in the second, because in the second at least some proportion of the genes would initially have had a narrower tissue expression, giving less time for the accumulation of Alu sequences. The expression modification by Alu hypothesis predicts the opposite result.

**Figure 4:**
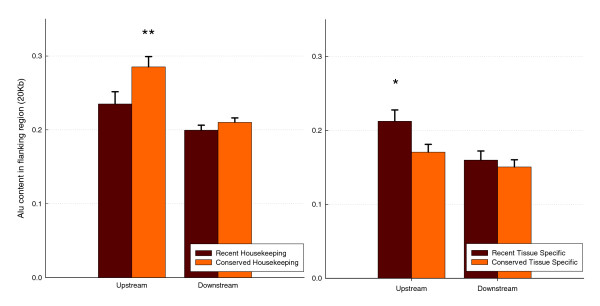
Alu content in flanking regions of recent expression profile modification and conserved housekeeping or tissue-specific genes. Each data subset of human housekeeping genes (expressed in 30 or 31 tissues of 31 in total) and tissue-specific genes (expressed in 1 or 2 tissues from 31 in total) was divided into two groups according to whether their mouse ortholog was a housekeeping or tissue-specific gene (if expressed in 30 to 31 or 1 to 2 tissues, respectively). The left panel shows human housekeeping genes for which the mouse counterparts are also housekeeping (orange columns) or tissue-specific instead (red columns). The right panel shows Alu content in tissue-specific human genes for which the mouse counterparts are also tissue specific or housekeeping instead. Stars represent significant differences in between the two groups with a P < 0.05 (*) and 0.01 (**) on a Students T-test.

Results of this analysis show that those genes that are housekeeping in both species indeed have a higher Alu content on both flanks, although this is only significant for the 5' region after Bonferroni correction (Student's *t*-test; upstream: *P *= 0.00278; downstream: *P *= 0.23845; Figure 4). Similarly, if the same test is applied to human tissue-specific genes, then those genes that are also tissue specific in mouse have significantly lower Alu content in their flanking regions than those genes that are broadly expressed in mouse (Student's *t*-test; upstream: *P *= 0.01231; downstream: *P *= 0.27760; Figure 4). A similar analysis was conducted for bodymap data, yielding similar results (see Materials and methods, below).

Based on these findings, we conclude that increased Alu sequences in flanking regions of housekeeping genes does not reflect modification of expression breadth by Alus. Instead, Alus accumulate in the vicinity of genes that already have greater breadth of expression, as expected under the marker model.

### Alu content is marginally related to estimates of transcription divergence

Having found that Alu enrichment around housekeeping genes does not appear to be the result of Alu-induced increased breadth of expression, we examined whether Alu insertions could be related to other measures of expression profile divergence between human-mouse ortholog gene pairs. For example, Alu insertions may induce changes not in the overall number of tissues where a gene is expressed but in the specific tissues where a gene is expressed. Alu insertions could also result in changes in expression intensity. These changes would not be picked up by comparing total number of tissues in which a gene is expressed. If Alus have contributed to expression evolution in primates, then we would expect that those genes with the highest Alu content would have diverged the most in terms of their gene activity.

**Figure 5:**
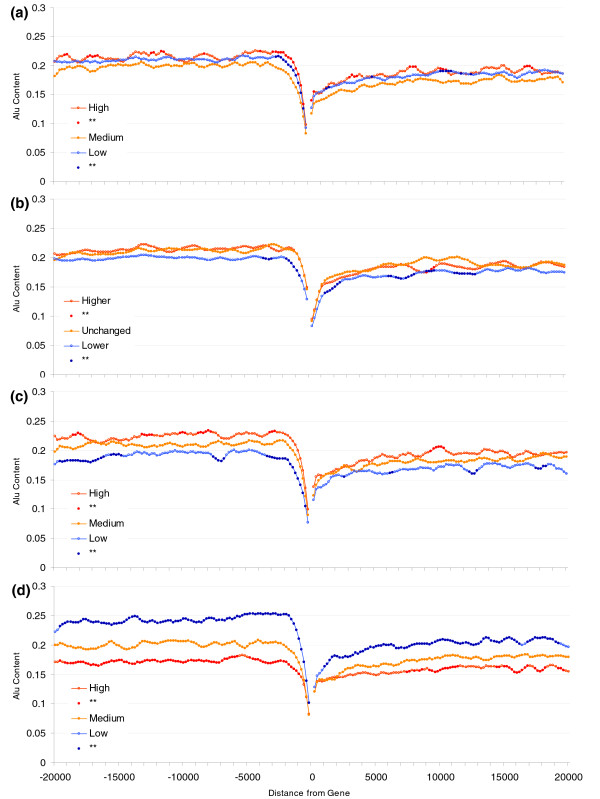
Alu content and expression divergence between human and mouse orthologous genes. **(a) **Number of switches from expressed to non-expressed; **(b) **ranked peak of expression difference; **(c) **expression intensity divergence estimated by using correlation coefficients as measure of distance; and **(d) **expression intensity divergence estimated by using Euclidean distances.

We first turned our attention to changes in the tissue distribution of gene expression by calculating the number of switches from expressed to nonexpressed between the two species for each tissue. We find weak and contradictory evidence; array data suggest no effect and bodymap data suggest a very weak effect (microarray/Bodymap [*n *= 11,275/8,179]; upstream: *r *= NS/0.048 [*P *NS for microarray and *P *< 0.001 for Bodymap]; downstream: *r *= NS/0.031 [*P *NS for microarray and *P *= 0.005 for Bodymap, but NS after Bonferroni correction]; Figure 5 and Additional data file 2).

We then looked at expression intensity, because it could still be the case that Alus sometimes cause expression increases/decreases while not changing the tissue in which a gene is expressed. We assessed changes in peak expression across all tissues and divergence by quantifying the differences in expression intensity in each tissue for each pair of orthologous genes. To compare peak expression between orthologous pairs, we used ranked peak expression, which allows comparison of data for human and mouse genes and smoothes out noise. (Note that this potentially misses subtle quantitative effects.). We find evidence for a weak relation with Alu content under one of the two expression data platforms (microarray [*n *= 11,275]; upstream: *r *= 0.038 [*P *< 0.001]; downstream: *r *= 0.024 [*P *= 0.02; not significant after Bonferroni correction]; for Bodymap data the relation was not significant; Figure 5 and Additional data file 2).

As for divergence in expression intensity profiles, we obtained two different measures to quantify the changes in expression intensity per tissue (correlation coefficients and Euclidean distances). These two measures examine whether Alus could be causing more subtle changes in expression intensity other than increased/decreased overall peak expression. We again find that Alu content is related to quantitative divergence for both the microarray dataset (correlation coefficients/Euclidean distances [*n *= 11,275]; upstream: *r *= -0.066/-0.096 [*P *< 0.001]; downstream: *r *= -0.033/-0.054 [*P *< 0.001]; Figure 5) and the Bodymap dataset (correlation coefficients/Euclidean distances [*n *= 8,179]; upstream: *r *= -0.057/-0.119 [*P *< 0.001 for both]; downstream: *r *= -0.026/-0.067 [*P *= 0.017 for correlation coefficient (not significant after Bonferroni correction) and *P *< 0.001 for Euclidean distance]; see Additional data file 2).

To examine whether these correlations could be explained by a shift in regional base composition, we examined whether the observed link between quantitative expression divergence and Alu persists after correcting for shifts in regional GC content between human and mouse. We find that this is not the case; the relation between Alu content and quantitative estimates of gene expression divergence remains significant after taking into account regional shifts in GC between the two species (correlation coefficients/Euclidean distances, microarray [*n *= 11,275]; upstream: *r *= -0.065/-0.089 [*P *< 0.001]; downstream: *r *= -0.036/-0.049 [*P *< 0.001]; Bodymap [*n *= 8,179]; upstream: *r *= -0.060/-0.116 [*P *< 0.001]; downstream: *r *= NS/-0.066 [*P *NS for correlation coefficient and *P *< 0.001 for Euclidean distance]).

In sum, both Bodymap and array data agree that Alu density correlates weakly with expression divergence. That the two datasets agree suggests that the correlations are not an artefact of expression platform. What is unclear is what it means. Most noteworthy in this context is the discrepancy in the direction of the relation with Alus between the two divergence measurements used. Higher Alu content is associated with lower *r *values and lower Euclidean distances. However, although low r values imply more divergence, lower Euclidean distances imply less divergence. So, are Alu associated with high or low divergence? Liao and Zhang [65] suggest that correlation coefficients as a measure of divergence would miss any linear changes in expression profiles, which might explain the rather weak relation with Alu content. If so, then we are then left to conclude that those genes with higher Alu content have diverged less from their mouse counterparts. This would be expected if Alu accumulate near to housekeeping genes and housekeeping genes have relatively stable expression profiles. Indeed, tissue-specific genes might be more likely to diverge neutrally in their expression rate, making this an attractive model. However, given that Alus might be related to higher divergence (as suggested by the correlation coefficient method), it would be unwise to suggest that this is in any manner a robust conclusion.

## Discussion

### Alus are markers of higher breadth of expression in primate genomes

Among all repetitive elements in the human genome, Alu sequences are unique in several respects. Apart from being the most common repetitive element, Alus are primate specific. Alu sequences are enriched in gene-dense regions [13], particularly in the vicinity of housekeeping genes [15,16]. This has prompted hypotheses for a widespread effect of Alu sequences in regulating gene expression [6,7,37] and hence controlling the morphologic characters of primates [6,7,12,37]. This is supported by evidence from only a few genes [6,19-26]. Our results, by contrast, show that Alu-mediated increases in expression breadth do not account for a major part of the difference found between primate and rodent transcriptomes as regards expression breadth. Moreover, their avoidance of transcriptional start sites argues strongly against their acting as CpG islands. Instead, the notion that Alu presence is a marker of expression breadth makes for a more parsimonious interpretation of the evidence.

What processes might account for Alu enrichment in the 5'-flanking regions of human housekeeping genes? There could be neutral and selectionist hypotheses. Several retroelements exhibit an open chromatin 5' insertion flanking region bias [56], which could provide a neutral hypothesis to, in part, explain the observed Alu pattern. However, Alus appear to insert preferentially in AT-rich regions rather than on GC-rich regions, where gene density is higher [1,60,61] (but see [62]), and so insertion bias alone is unlikely to account for all features of the skewed distribution. The reasons for the shift from AT-rich regions, where young Alus are more commonly found, to the GC-rich regions, where older Alus are concentrated, are a matter of debate. Some authors have proposed that neutral processes, such as variations in rates of recombination [1,13,66-72] or changes in insertion preferences [72], might account for the observed distribution. Eller and coworkers [17] suggest, for example, that illegitimate recombination between linked Alu can cause deletions that remove not just the Alu but intervening sequence as well. In some genomic domains, such deletions might be more likely to be neutral rather than deleterious. This might explain why Alus end up being common in gene-dense regions, because in such regions a deletion is more likely to be deleterious. Perhaps with a higher density of control elements 5' than 3' of genes, such a model might also go some way toward explaining the observed somewhat greater 5' than 3' enrichment. Alternative selectionist models to that of Alus as modifiers of gene expression breadth are also possible. For example, one might suppose that Alus are situated in chromatin domains that permit their expression should it be required, for example under stressful conditions [38-41]. It has, however, been pointed out that the rate of fixation of Alus in GC-rich regions is so slow that it might better be explained by neutral processes [67].

### Alus flanking housekeeping genes partly explain their relation with functional categories

How then might we explain other curious features of the distribution of Alus, such as their association with genes of particular functional classes? Two studies have reported that Alu sequences are found at different frequencies in genes that serve different functions in the cell. One of the studies was limited to genes found in chromosomes 21 and 22, and focused only on Alus residing within genes [18]. The second study was genome wide in scope and focused on the Alus present at the 5' flanking region of genes [37]. Both studies showed that genes associated with certain gene functions have significantly more Alus, either within the gene or in their flanking regions. Polak and Domany [37] appear to assume that most of the variation observed in Alu frequencies linked to different cell functions is related to the fact that Alu sequences contain transcription factor binding sites.

**Figure 6:**
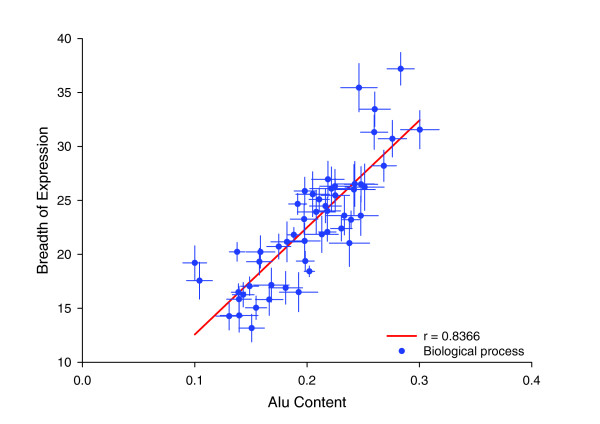
Average Alu content and breadth of expression for genes serving different biologic processes. Average Alu content and breadth of expression was obtained for genes associated with different biologic processes (according to the Gene Ontology database; only categories with more than 100 genes were taken into account). Each point in the graph represents genes associated with a particular biologic process. Error bars are standard errors for each group of genes in terms breadth of expression (vertical bars) and Alu content (horizontal bars). Regression line is shown in red.

Might the marker model also account for such biases? It is possible that broadly expressed genes are skewed as regards their cellular functions, in which case an incidental correlation with Alu content would be expected. Indeed, we found that there is a significant association between expression breadth and gene function (data not shown). We calculated the average breadth of expression and Alu content in the upstream flanking regions of genes associated with different biologic processes. Figure 6 shows that those biologic processes with the highest average Alu content in their flanking regions are also associated with a higher average breadth of expression (*r *= 0.836 [*P *< 0.0001], *n *= 53 processes; Table 1). This suggests that skews in the sorts of genes serving particular cellular functions enriched for Alus can be, at least in part, accounted for by the fact that Alus are housekeeping gene markers.

**Table 1:** Average Alu and breadth composition for human genes associated with different biological processes

Biological process	Gene number	Breadth		Alu	
		
		Mean	SD	Mean	SD
Protein biosynthesis	255	37.2	24.11	0.2833	0.1932
Ubiquitin-dependent protein catabolism	111	35.45	23.57	0.2462	0.1707
Intracellular protein transport	207	33.45	22.93	0.2602	0.2001
Nuclear mRNA splicing, via spliceosome	163	31.56	22.56	0.3004	0.2186
Protein folding	201	31.33	22.5	0.2597	0.1716
Protein transport	193	30.72	23.46	0.2759	0.1749
Ubiquitin cycle	270	28.21	23.77	0.2683	0.1826
Protein amino acid dephosphorylation	154	26.96	20.75	0.2186	0.1792
Protein modification	104	26.53	21.2	0.2425	0.1772
Regulation of progression through cell cycle	185	26.5	22.25	0.248	0.2014
Cell cycle	186	26.32	21.2	0.2248	0.1852
Protein complex assembly	108	26.23	22.33	0.2513	0.1806
Cell motility	105	26.1	20.57	0.2217	0.1976
Transcription	101	26.01	23.31	0.2417	0.1906
Electron transport	317	25.87	22.7	0.198	0.1703
Negative regulation of cell proliferation	122	25.57	22.99	0.205	0.1657
Small GTPase mediated signal transduction	194	25.47	21.47	0.2251	0.1775
Intracellular signaling cascade	406	25.1	21.65	0.2108	0.1811
Metabolism	440	24.68	20.43	0.1916	0.1681
Carbohydrate metabolism	191	24.48	21.51	0.2164	0.1956
Apoptosis	260	24.03	20.43	0.218	0.1906
Cytokinesis	111	23.94	21.26	0.2082	0.1773
Regulation of transcription from RNA polymerase II promoter	150	23.59	21.86	0.2332	0.1938
DNA repair	132	23.58	21.06	0.2478	0.179
Cell proliferation	223	23.26	20.79	0.1974	0.1847
Protein amino acid phosphorylation	611	23.21	20.35	0.2391	0.1881
Protein ubiquitination	346	22.39	21.55	0.2305	0.1772
Transport	580	22.07	20.49	0.2184	0.1862
Transcription from RNA polymerase II promoter	171	21.85	22.01	0.2131	0.192
Signal transduction	896	21.81	20.21	0.1882	0.1702
Lipid metabolism	156	21.24	20.66	0.1978	0.1732
Cell surface receptor linked signal transduction	126	21.16	20.82	0.1821	0.1603
DNA replication	103	21.08	21.71	0.2377	0.1818
Immune response	266	20.72	18.79	0.1748	0.1743
Cell adhesion	439	20.23	18.16	0.1377	0.1425
Inflammatory response	174	20.22	19.76	0.1585	0.1693
Proteolysis	474	19.38	19.72	0.1984	0.1739
Nervous system development	242	19.32	19.21	0.1575	0.1769
Sensory perception of smell	132	19.21	18.16	0.1	0.1211
Regulation of transcription, DNA dependent	1681	18.42	20.2	0.2021	0.1877
Homophilic cell adhesion	117	17.56	18.49	0.1042	0.1238
Chemotaxis	105	17.15	16.11	0.1682	0.162
Development	474	17.05	18.67	0.1488	0.1737
Muscle development	127	16.9	17.06	0.1809	0.1695
Spermatogenesis	107	16.5	18.73	0.1925	0.1767
G-protein-coupled receptor protein signaling pathway	547	16.49	18.41	0.1388	0.1478
Ion transport	231	16.26	17.18	0.1437	0.1471
Synaptic transmission	190	15.84	16.34	0.1394	0.1516
Visual perception	168	15.82	19.07	0.1663	0.159
Cell-cell signaling	269	15.05	17.83	0.1546	0.1559
Phosphate transport	104	14.34	15.81	0.1395	0.1712
Potassium ion transport	168	14.27	16.54	0.1306	0.1533
Cation transport	185	13.17	17.41	0.1506	0.1541

Mean and standard deviation (SD) for breadth and Alu content in the upstream region 2 to 6 kilobases away from transcription starting site are shown for genes associated to each biological process. Only Gene Ontology (GO) categories containing more than 100 genes from our sample were included in the analyses. Note that GO categories are not mutually exclusive.

In a related vein, because housekeeping genes tend to be slow evolving [73,74], we might also expect Alu to reside near to genes with low rates of protein evolution. This is indeed the case, albeit only marginally so; K_a _values are correlated to Alu content in 5' flanking region (*r *= 0.051 [*P *< 0.001], *n *= 11,896), but not with downstream Alu content. The synonymous substitution rates are not significantly related to Alu content in flanking regions, suggesting that point mutation and Alu insertions/fixations/preservation are not related processes.

## Conclusion

In summary, we find that there is Alu enrichment at flanking regions of housekeeping genes and that previously reported enrichment for highly expressed genes is a byproduct of the co-variance between breadth and peak expression. This enrichment is not explained by the relation of both breadth of expression and Alu density to regional GC content. The results from the comparative transcriptomics analyses presented here provide no evidence that Alu sequences have boosted breadth of expression of adjacent genes during evolution of the primate transcriptome. Our results suggest instead that Alus just tend to accumulate in the vicinity of housekeeping genes; the marker model is then more parsimonious. Alus are related to other measures of expression divergence but the results are contradictory; by one measure they are associated with greater divergence, whereas possibly the more robust measure suggests that they are associated with less divergence.

## Materials and methods

### Sequence analysis

Upstream and downstream flanking regions were downloaded for 20,490 human (20 kb) and 18,409 mouse (10 kb) genes from Ensembl [75]. Alu sequences were then identified and masked using RepeatMasker [76] for the human sequences. Masked sequences were divided using a sliding window approach into 1,000 bp bins moving in steps of 200 bp. Alu content (proportion of the bin occupied by masked sequence) and GC content (for the masked and unmasked sequences) were calculated for each bin. Mouse flanking sequences were also analyzed through a sliding window approach to calculate GC content. The automation of repeat masker and the sliding window analysis were performed using a script developed by LBO and is available upon request.

### Expression data

Quantitative estimates of gene activity were obtained from Su and colleagues [44] for mouse and human genes. All probes matching to the same gene were averaged. Data were available for 63 tissues obtained from healthy human adults. Corresponding mouse expression data were available for 26 tissues from the same source [44]. Two indices of gene activity were obtained - peak expression in any given tissue and breadth of expression, or the number of tissues in which a gene is expressed - for a total of 15,538 genes. Quantitative estimates of gene expression were obtained by normalizing the original signal values. Peak expression was the highest expression in any given tissue was taken for each gene. For breadth two procedures were used to estimate whether a gene was being expressed at a given tissue, the first index simply takes the absence/presence (AP) calls provided (see Su and coworkers [44] for details). However, this was not ideal because there were huge variations in the total number of genes expressed from tissue to tissue. Our second approach required normalization such that the S values for each tissue would total 1,000,000. We then applied a cut-off value of 50 (which corresponds in the average sample to the 200 cut-off value as suggested by Su and coworkers [44]) to determine whether a gene is expressed or not. The correlation between the two measures was high (*r *= 0.714) and both were similarly related to Alu content, although the normalized values were a significantly better predictor of Alu content. All analyses in this report use a measure of breadth of expression derived from the normalized quantitative values, but similar results were obtained when using the AP calls.

SAGE data were also obtained for human genes. Only normal tissue libraries were included in analyses (corresponding to 35 tissues). All libraries corresponding to the same tissue were pooled together. Best matching tag for each gene was obtained from the SAGE genie website of the National Center for Biotechnology Information [45]. We then normalized all libraries to 10,000 tags, and in any given tissue genes with counts lower that 1 were considered not expressed. Peak and breadth of expression indices were then calculated for each gene. Note that the analyses were also performed by using the best gene for each tag annotation. In this case, all tags matching to the same gene were averaged together. Similar results were obtained in the best-tag-per-gene analyses.

Expressed sequence tag Bodymap data [46] were obtained for 37 human and mouse normal tissues in the form of count per million. Unigene IDs were matched to Ensembl gene IDs. Breadth and peak expression were calculated for human and mouse genes.

### Correction of parameters by regression analysis

To correct for the effect of GC content on Alu content, we took the residuals from a regression analysis for each 1 kb window of Alu content predicted by GC content in the same region. Linear fits were used unless polynomial fits yielded significantly better fit. The correction was performed using values of GC content in both masked (for Alus) flanking sequences. A similar procedure was used to correct for the effect on expression of coding sequence divergence. Measures of distance used included non synonymous substitution rate, synonymous substitution rate, and non synonymous/synonymous rates of substitution (from the Ensembl website [75]).

### Regional Alu content similarity

To correct for the similarity in Alu content in 5' and 3' flanking regions, we regressed the content for each window of 1,000 bp in the 5' flanking region with the average Alu content in the 3' flanking region as the predictor. Alu content in 5' flanking region was the expressed as the residual values of these regressions. The opposite was done to correct for the Alu content local similarity in the 3' flanking region.

### Human-mouse gene orthology

A sample of 11,896 homolog pairs of human and mouse genes was gathered from the Ensembl website [75]. All genes with more than one homolog match were eliminated from further analysis. Measures of coding sequence divergence (dn, ds, and dn/ds) were also obtained from the same source.

### Human-mouse expression divergence

Quantitative estimates of gene activity for mouse genes were obtained from Su and colleagues [44]. All probes matching to the same gene were averaged together. Genes with no unique homolog counterpart were eliminated. Only tissues for which libraries were present in both species were used in gene activity divergence assessment (32 tissues for healthy adults). All samples were then normalized to make possible the comparison between mouse and human counterparts. Two types of expression divergence were obtained. 'Difference' refers to the difference in the indices of peak/breadth between human and mouse. For breadth, 'difference' simply refers to the difference in the number of tissues both genes are expressed. For quantitative expression, peak values for both mouse and human genes were ranked and then difference in ranks was then calculated. The second set, expression 'divergence' indices, relates in the case of breadth of expression to the sum of switches from expressed to nonexpressed changes in expression for each tissue. In the case of quantitative expression, the divergence between human mouse orthologs was calculated after correcting the expression values using a scaling method described by Liao and Zhang [65]. Correlation coefficients and Euclidean distances were then obtained from the scaled expression vectors for every pair of orthologous genes. Quadratic regression coefficients were used in assessing the relation of divergence indices and Alu content as relationships do not always follow a linear trend (see Additional data file 3 for histograms).

Divergence estimates from the Bodymap dataset [46] were obtained for quantitative and binomial measures of expression profiles in a manner similar to that used for the microarray data. Breadth and peak differences were calculated for each human-mouse orthologous pair. Breadth divergence was calculated as the number of switches from expressed to nonexpressed and *vice versa*. Quantitative divergence was calculated as the correlation coefficient and Euclidean distances over counts per million and after scaling data [65] (See Additional data file 4 for histograms).

### Housekeeping tissue-specific analysis

In the case of Bodymap data, there were very few gene pairs in which the human copy was housekeeping and the mouse counterpart was tissue specific and *vice versa*. We therefore decided to take the top and bottom 5% of the distribution of human breadth of expression. If the 5% limit left out some of the genes expressed in the same number of genes as the last gene selected, then those were included as well. For each group we compared Alu content for the top and bottom 5% of the genes in the distribution in terms of mouse breadth. We found that although there is no significant differences in human breadth of expression between the groups, Alu content was higher in those genes in which the mouse ortholog had a higher breadth of expression (Student's *t*-test, human high breadth; upstream: *P *= 0.00931; downstream: *P *= 0.04558 [*n *= 81 *Homo sapiens *high - *Mus musculus *high, and *n *= 20 *Homo sapiens *high - *Mus musculus *low]; human low breadth; upstream: *P *= 0.005593; downstream: *P *= 0.570325 [*n *= 25 *Homo sapien *low - *Mus musculus *high, and *n *= 29 *Homo sapiens *low - *Mus musculus *low]).

### Gene function

Biologic processes to which each gene is associated were obtained from the Gene Ontology database. Each gene can be associated with more than one gene function. Only those categories that contained more than 100 genes from our sample were included in the analysis (Table 1).

## Abbreviations

AP, absence/presence; bp, base pairs; kb, kilobase; NS, not significant; SAGE, Serial Analysis of Gene Expression; TSS, transcriptional start site.

## Authors' contributions

AOU conceived of the project. AOU and LDH designed the experiments. AOU and LBO obtained data and performed analyses. AOU and LDH wrote the paper.

## Additional files

The following additional data are available with the online version of this paper. Additional data file 1 shows Alu content in flanking regions of human genes (20 kb) and expression profiles (SAGE and Bodymap data). Additional data file 2 shows Alu content and expression divergence between human and mouse orthologous genes for Bodymap data. Additional data file 3 shows the histograms for all measures of expression divergence between human and mouse for microarray data. Additional data file 4 shows histograms for all measures of expression divergence between human and mouse for Bodymap data.

## Supplementary Material

Additional data file 1 Groups represent the 20% most highly, least highly, and medium expressed genes for peak and breadth. Points for 'high' and 'low' groups significantly different from medium expression levels (Student's *t*-tests using Bonferroni correction) are represented by closed circles. Each point represents the Alu content in sliding windows of 1 kb (moving 200 bp at a time). Click here for file

Additional data file 2 From top to bottom, each panel shows the following: difference in breadth of expression; number of switches from expressed to non-expressed; ranked peak of expression difference; expression intensity divergence estimated by using correlation coefficients as measure of distance; and expression intensity divergence estimated by using Euclidean distances. Click here for file

Additional data file 3 From left to right and top to bottom: differences in total breadth; number of switches from expressed to non-expressed; differences in peak of expression; quantitative expression divergence, assessed as Euclidean distances between orthologous pairs; and quantitative expression divergence, assessed as correlation coefficients between orthologous pairs. Click here for file

Additional data file 4 From left to right and top to bottom: differences in total breadth; number of switches from expressed to non-expressed; differences in peak of expression; quantitative expression divergence assessed as correlation coefficients between orthologous pairs; and quantitative expression divergence, assessed as Euclidean distances between orthologous pairs. Click here for file
