# Supplementary figures and images for: Do Alu repeats drive the evolution of the primate transcriptome?
Source: Genome Biol. 2008 Feb 1;9(2):R25. doi: 10.1186/gb-2008-9-2-r25 (PMC2374697; doi:10.1186/gb-2008-9-2-r25)

## SAGE

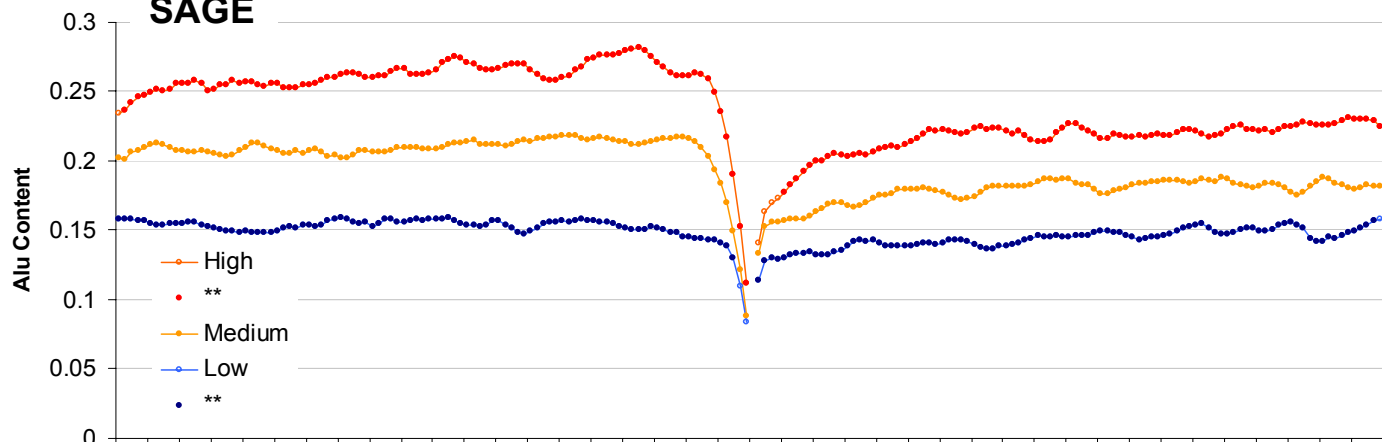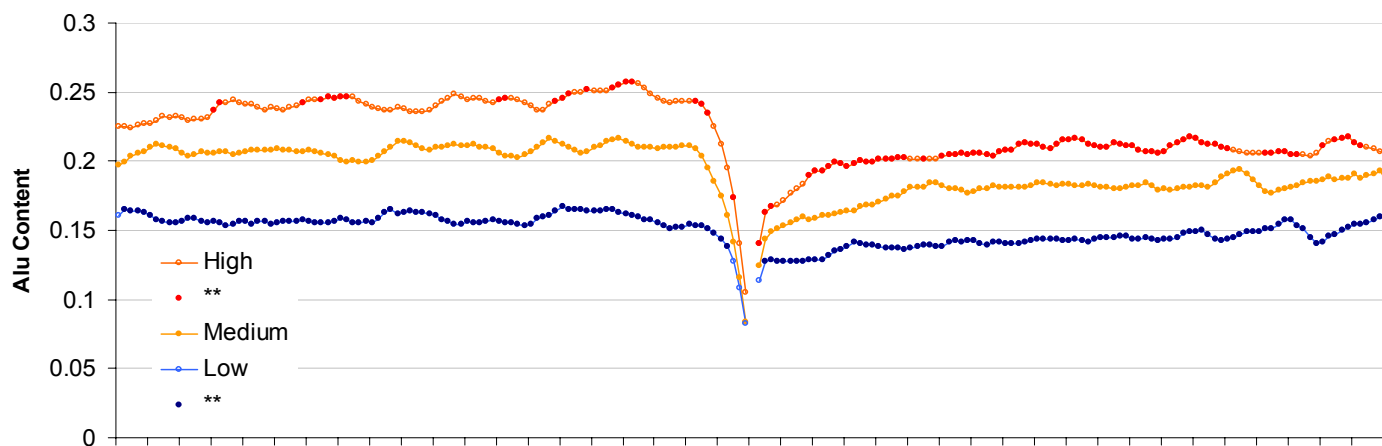

## Bodymap

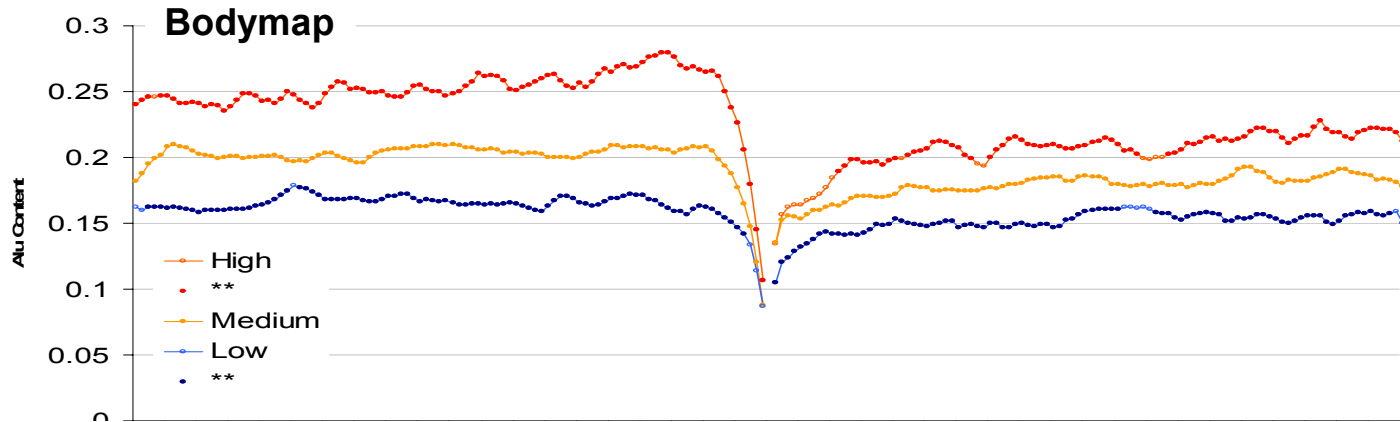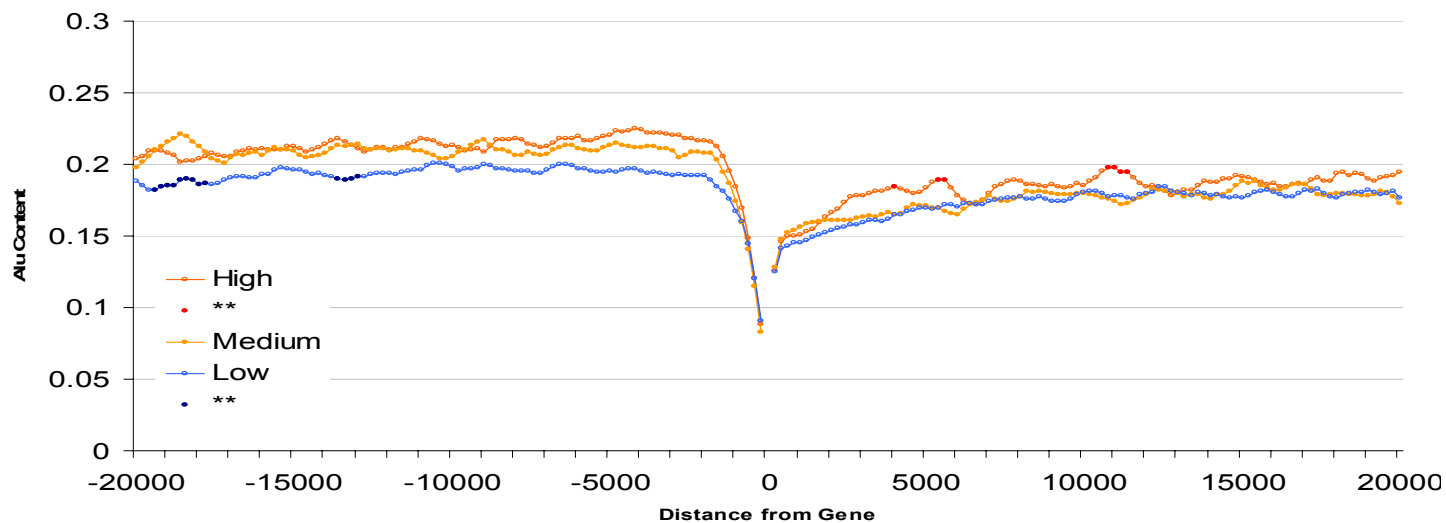

Supplement: Additional data file 1 — Groups represent the 20% most highly, least highly, and medium expressed genes for peak and breadth. Points for 'high' and 'low' groups significantly different from medium expression levels (Student's t-tests using Bonferroni correction) are represented by closed circles. Each point represents the Alu content in sliding windows of 1 kb (moving 200 bp at a time). [file gb-2008-9-2-r25-S1.pdf]

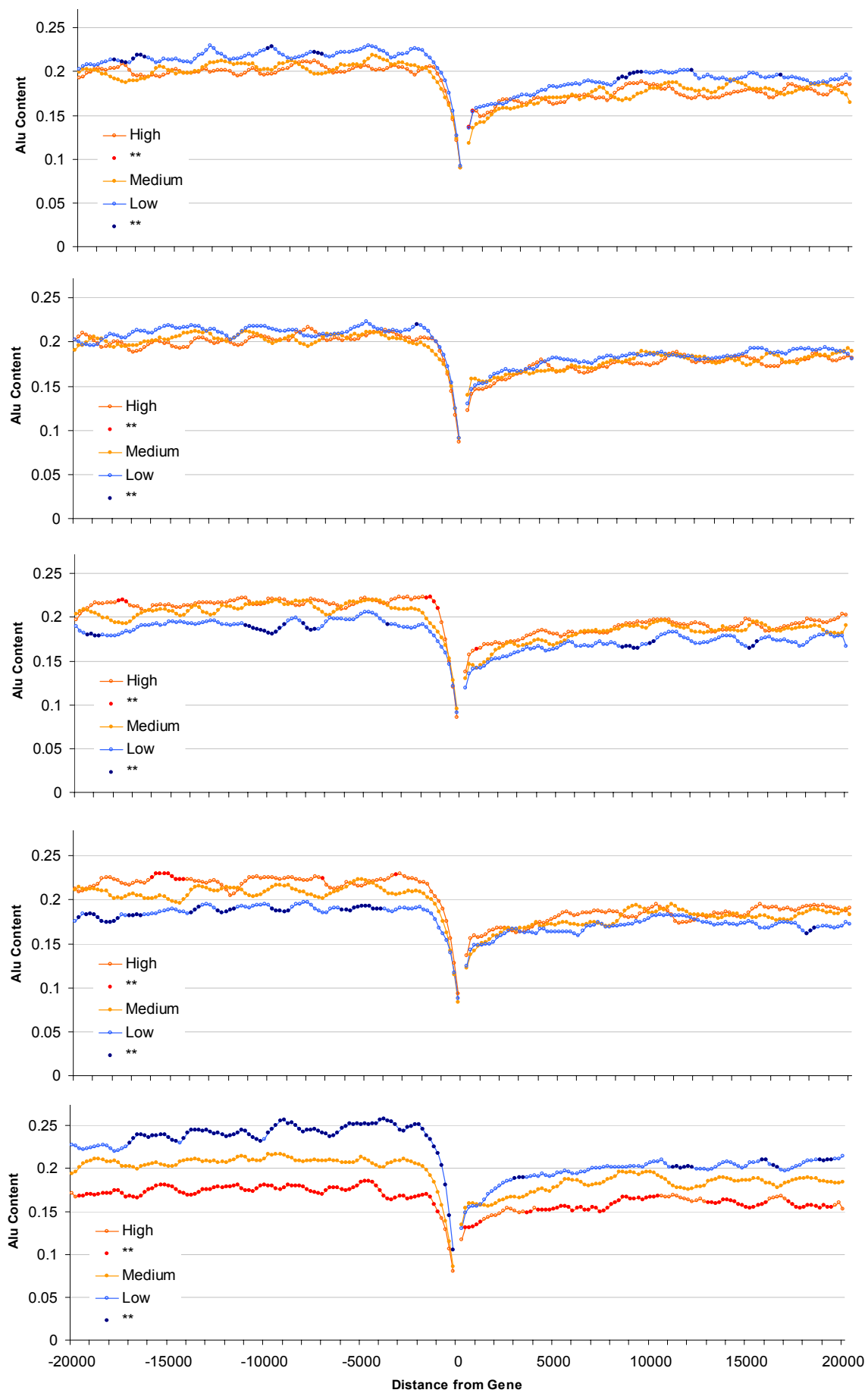

Supplement: Additional data file 2 — From top to bottom, each panel shows the following: difference in breadth of expression; number of switches from expressed to non-expressed; ranked peak of expression difference; expression intensity divergence estimated by using correlation coefficients as measure of distance; and expression intensity divergence estimated by using Euclidean distances. [file gb-2008-9-2-r25-S2.pdf]

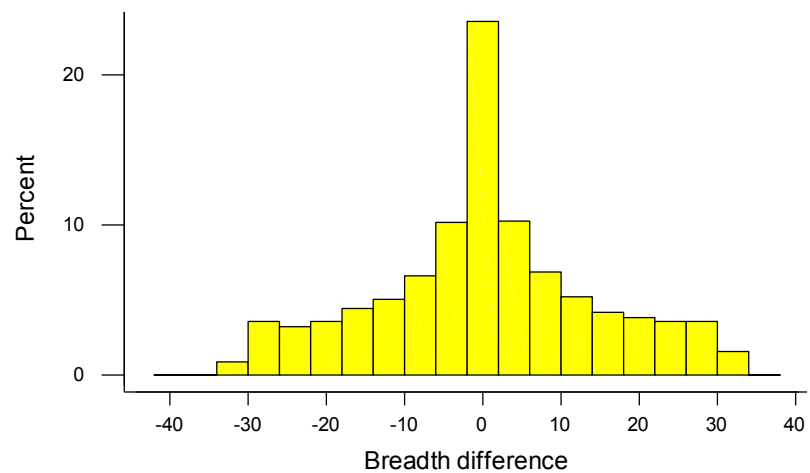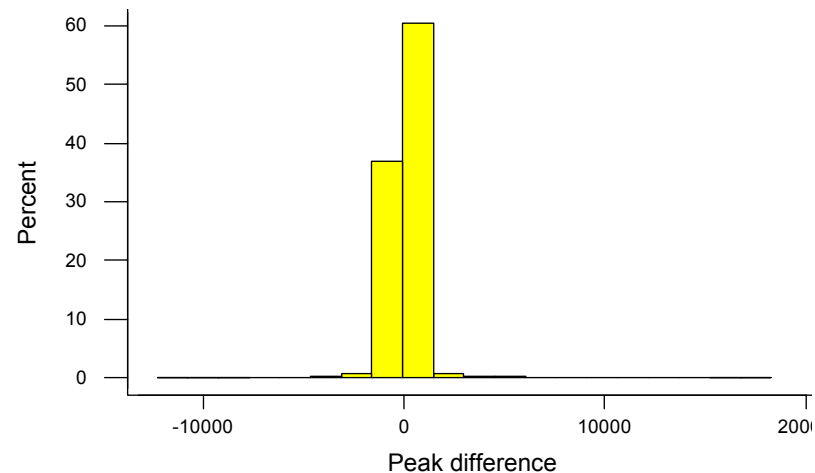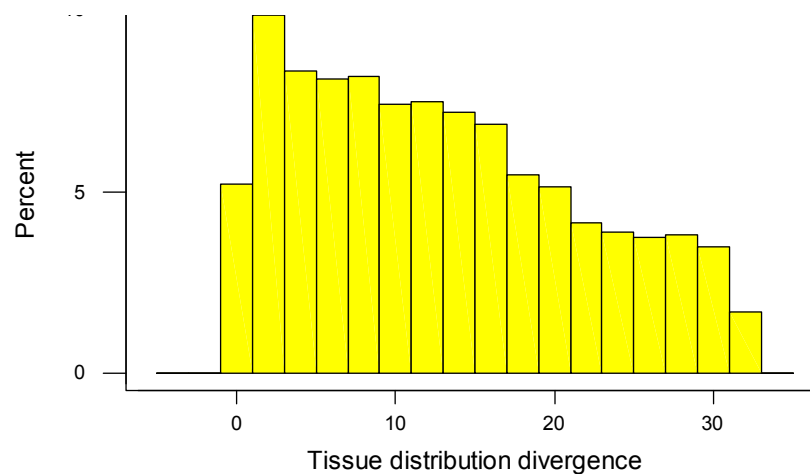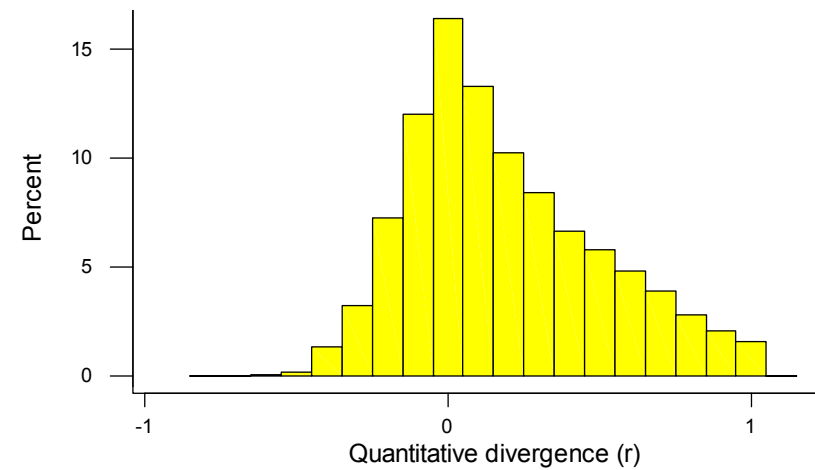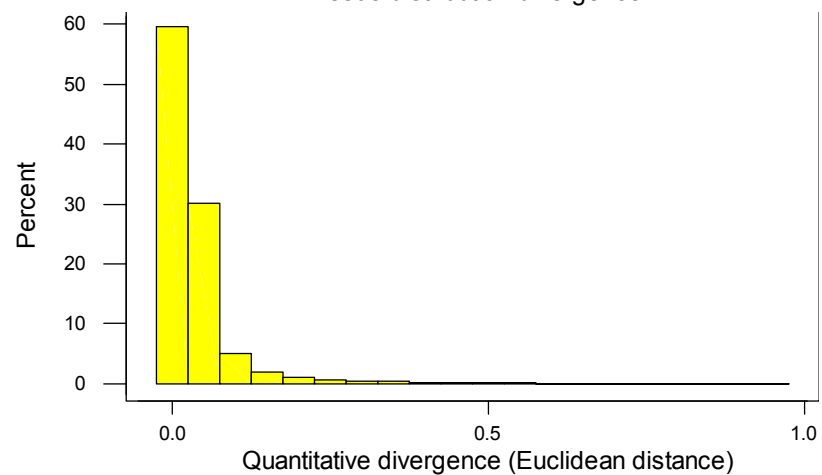

Supplement: Additional data file 3 — From left to right and top to bottom: differences in total breadth; number of switches from expressed to non-expressed; differences in peak of expression; quantitative expression divergence, assessed as Euclidean distances between orthologous pairs; and quantitative expression divergence, assessed as correlation coefficients between orthologous pairs. [file gb-2008-9-2-r25-S3.pdf]

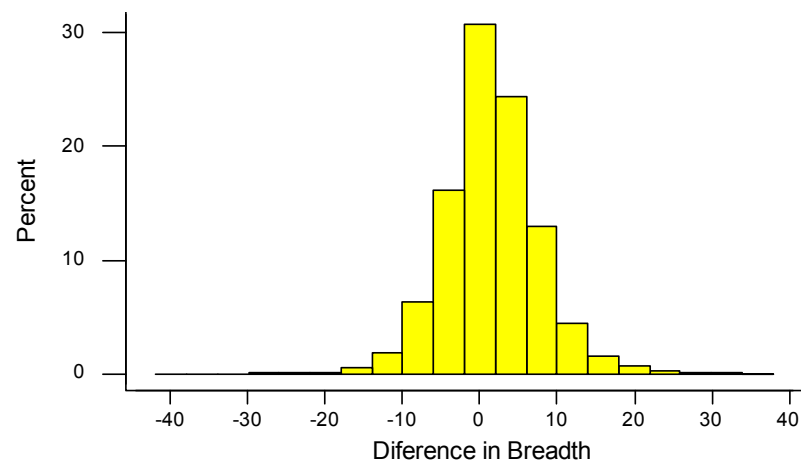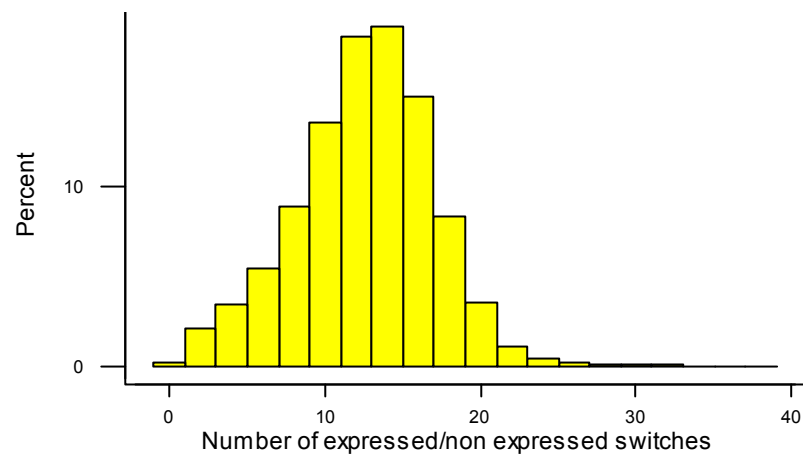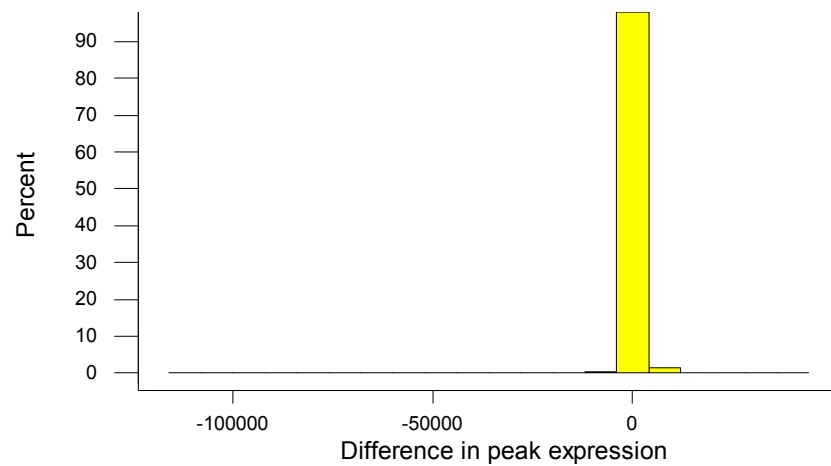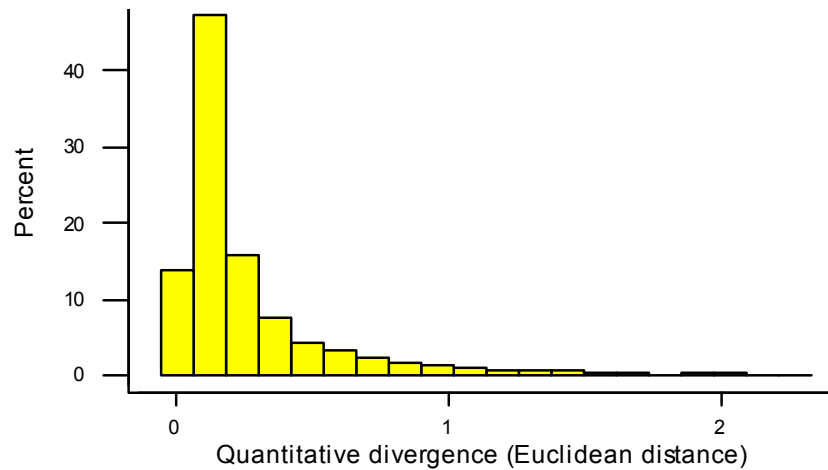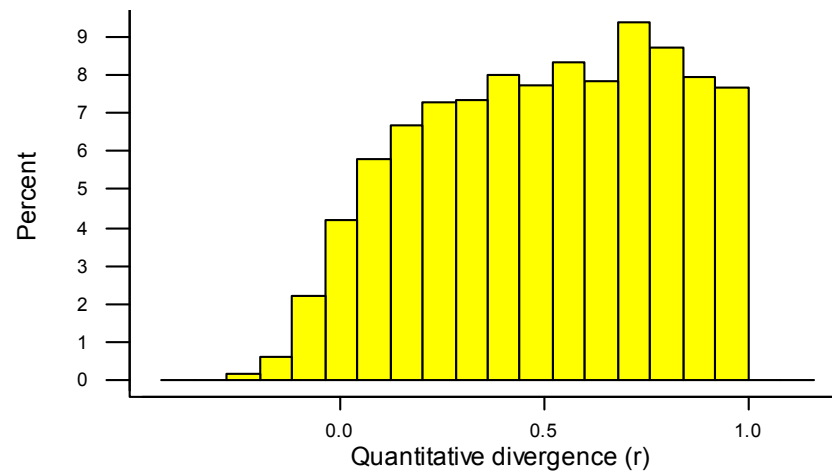

Supplement: Additional data file 4 — From left to right and top to bottom: differences in total breadth; number of switches from expressed to non-expressed; differences in peak of expression; quantitative expression divergence assessed as correlation coefficients between orthologous pairs; and quantitative expression divergence, assessed as Euclidean distances between orthologous pairs. [file gb-2008-9-2-r25-S4.pdf]
